# Complete Remission after Sequential Therapy of Drug Eluting Beads Transarterial Chemoembolization and Liver Resection in Large Solitary Nodule Hepatocellular Carcinoma

**DOI:** 10.1155/2017/3682614

**Published:** 2017-09-27

**Authors:** Juferdy Kurniawan, Andri Sanityoso Sulaiman, Sahat Basana Romanti Ezer Matondang, Toar Jean Maurice Lalisang, Ening Krisnuhoni, Steven Zulkifly

**Affiliations:** ^1^Division of Hepatobiliary, Department of Internal Medicine, Faculty of Medicine, Universitas Indonesia, Cipto Mangunkusumo National General Hospital, Jakarta, Indonesia; ^2^Department of Radiology, Faculty of Medicine, Universitas Indonesia, Cipto Mangunkusumo National General Hospital, Jakarta, Indonesia; ^3^Department of Surgery, Faculty of Medicine, Universitas Indonesia, Cipto Mangunkusumo National General Hospital, Jakarta, Indonesia; ^4^Department of Pathology Anatomy, Faculty of Medicine, Universitas Indonesia, Cipto Mangunkusumo National General Hospital, Jakarta, Indonesia

## Abstract

Hepatocellular carcinoma (HCC) is the fifth most prevalent and the second highest cause of death among cancer. The treatment of large solitary nodule HCC is still challenging. Transarterial chemoembolization (TACE) and liver resection are two modalities of therapy in HCC management. However, recurrence rate from each therapy is relatively high. We report a case of 46-year-old man diagnosed with large solitary nodule HCC, who was treated with drug eluting bead TACE (DEB-TACE) prior to liver resection. Studies about this combination are still limited and showed various results.

## 1. Introduction

Transarterial chemoembolization (TACE) is a treatment of choice in hepatocellular carcinoma BCLC Stage B [1, 2]. Drug eluting beads-TACE (DEB-TACE) is a relatively new drug delivery embolization and developed to optimize the delivery of chemotherapeutic agents with minimal systemic toxicity [3, 4]. Complete response (CR) or partial response (PR) after TACE is very dependent on treatment regimen, the amount, and size of the tumor [5]. Large HCC nodule (size ≥ 5 cm) had a lower CR percentage. Nodule diameter < 5 cm had 95% CR after first DEB-TACE. Meanwhile, only 13% CR had been found for diameter ≥ 5 cm [6].

Liver resection is highly recommended for HCC BCLC Stage A, with single nodule with diameter < 5 cm or multiple nodules (up to 3 nodules) with each diameter < 3 cm (Milan Criteria) [1, 2]. However, many studies reported that liver resection is beneficial for single nodule HCC, without restriction of tumor size. Liver resection of HCC multiple nodules has been associated with lower 5-year survival rates [7, 8]. The median time from resection to recurrence is different from each study. The shortest and longest duration for recurrence are 22 months and 34 months, respectively, after primary resection [9–11].

Lim et al., in 2014, reported that solitary HCC with tumor size > 5 cm was not independent predictors of poor overall survival. The 5-year overall survival and recurrence-free survival rates of size between 5 and 10 cm were 58% and 26%, respectively, and size > 10 cm 53% and 24%, respectively [12]. Retrospective analysis of liver resection for large solitary HCC by Zhao et al. in 2016 showed that 5-year overall survival and disease-free survival were 43% and 47%, respectively [13].

Zhao et al. also reported the postoperative complications were found in 21% patients, including ascites (21.21%), transient hepatic dysfunction (9.9%), bile leakage (7.7%), and liver failure (4.4%). Thirty-day mortality after resection was 2.2% due to liver failure [13]. Recent study by Chen et al. compared the postoperative complications between solitary HCC < 10 cm and ≥10 cm. Total complications in HCC ≥ 10 cm were 39.1% and higher than HCC < 10 cm (29.8%). Liver failure occurred in 1% (2 patients) and 4% (1 patient), respectively [14].

There are limited studies about postsurgical complication of liver resection of large solitary HCC after TACE. Recent study reported postoperative complications were found in 59.2% (29 patients), including bile leakage (2 patients) and gastrointestinal hemorrhage (1 patient). The alteration of liver function was recovered in 1 month [15].

## 2. Case Presentation

**Figure 1:**
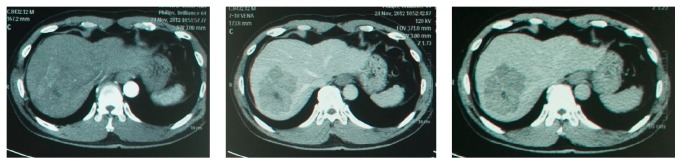
Arterial, venous, and delayed phase of Abdominal CT Scan after diagnosis (November 2012).

A man, 46 years old, came to his gastroenterohepatology consultant with chief complaint of abdominal pain on the right upper quadrant. He was diagnosed with hepatocellular carcinoma and chronic hepatitis B. The patient then underwent the 3-phase Abdominal CT Scan examination in November 2012 (Figure 1). The radiologic examination findings revealed an 8 cm solitary nodule HCC in segment 7. He was classified into BCLC Stage B with Child Pugh (CP) score A. The drug eluting bead (DEB) TACE procedure was then planned for the patient. He also received a lamivudine 100 mg once daily for chronic hepatitis B.

**Figure 2:**
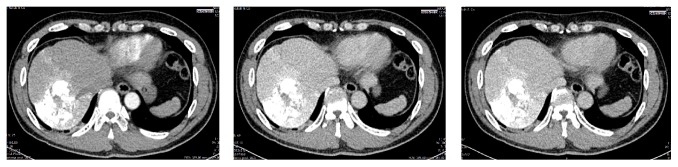
Arterial, venous, and delayed phase of Abdominal CT Scan after first DEB-TACE (January 2013).

**Figure 3:**
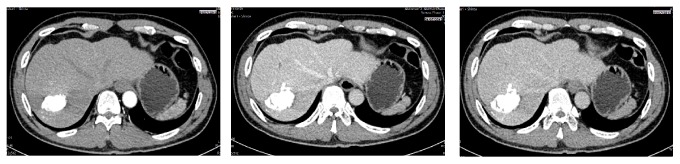
Arterial, venous, and delayed phase of Abdominal CT Scan after second DEB-TACE (May 2013).

The first DEB-TACE was performed in January 2013. Evaluation of the tumor was assessed by using 3-phase Abdominal CT Scan a month later. It showed minimal hypervascular lesion from middle hepatic artery (Figure 2). DEB-TACE was planned for the patient for the second time. Second DEB-TACE was performed 4 months later in May 2013 and 3-phase Abdominal CT Scan after TACE showed reduction of tumor size to 3 × 4 cm (Figure 3).

**Figure 4:**
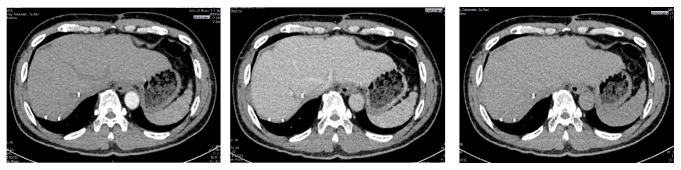
Arterial, venous, and delayed phase of Abdominal CT Scan 4 months after surgical resection (February 2014).

**Figure 5:**
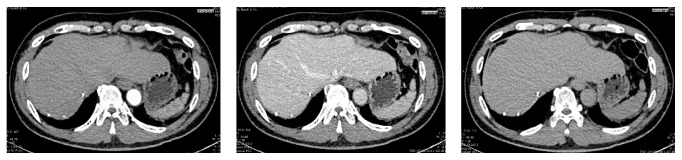
Arterial, venous, and delayed phase of Abdominal CT Scan 3 years after surgical resection (November 2016).

In November 2013, liver resection was performed in segments 6, 7, and 8 of the liver. The tissue was sent to pathology anatomy department for histopathological examination. The microscopic findings of resection showed HCC grade III. Radiographic examination of 4 months after liver resection showed no abnormality (Figure 4). Three years after resection, the patient underwent the Abdominal CT Scan examination and no tumor was found. (Figure 5).

## 3. Discussion

The research about TACE prior to liver resection is very limited. Small trial by Gerunda et al., in 2000, compared 20 HCC patients who underwent resection to TACE prior to resection. Early recurrence (<24 months) and late recurrence (>24 months) were found higher in resection alone group, with 59% and 10% compared to TACE and resection, with 20% and 10%, respectively [16].

Another study reported there are difference of 1-, 2-, and 5-year overall survival rates between the TACE prior to surgery and resection alone group (*p* = 0.11). Contrary to previous study, the 1-, 2-, and 5-year recurrence-free interval were higher in resection alone (97%, 83%, and 45%, resp.) compared to TACE-surgery group (58%, 36%, and 7%, resp.) with *p* = 0.01 [17]. TACE procedure induced tumor downstaging or necrosis and hypothesized to be associated with improvement of disease-free survival. The higher recurrence rate in TACE-surgical group was especially found in patients with initial resectable HCC. It was suggested that preoperative TACE might induce incomplete necrosis, resulting in hematogenous spread of residual tumor cells after liver resection, and caused recurrences [17, 18].

However, recent study in 2017 compared TACE prior to surgery (49 patients) and TACE alone in large/multifocal HCC (61 patients). All of the patients were classified in BCLC Stage B. The mean initial tumor in TACE + surgery and TACE group is 7.22 ± 3.18 cm and 6.80 ± 3.35 cm, respectively. However, the number of tumors was not limited to solitary tumor. The 1-, 2-, and 3-year overall survival rates in TACE + surgery group were 89.8%, 79.4%, and 59.1%, respectively, and in TACE alone were 75.1%, 61.5%, and 15.1%, respectively. In univariate analysis, solitary tumor was associated with higher overall survival, with *p* = 0.012 [15].

DEB-TACE has been found to be more effective for large nodule (>5 cm) compared to cTACE. In subgroup analysis of Asian patients who received DEB-TACE with size > 5 cm, DEB-TACE was reported to have significantly higher objective response compared to cTACE. The largest tumor size included in this study was 12 cm. Around 16.3% and 66.6% patients with large tumor size achieved CR and PR, respectively, in DEB-TACE group [16].

However, there are no publications that studied about the efficacy of DEB-TACE prior to surgery in large solitary nodule HCC. This case report presents a complete remission after 3 years of solitary large nodule of HCC with sequential therapy of DEB-TACE and liver resection. However, larger studies with longer duration are needed to find the efficacy, survival rates, and tumor recurrence.

For conclusion, the treatment of HCC with solitary large nodule is still challenging for clinicians. HCC patients who underwent TACE before resection have better overall survival, but it might have higher risk for recurrence compared to resection alone. TACE is safe procedure and effective for large HCC. DEB-TACE prior to liver surgery can be suggested for therapy of large solitary nodule of HCC.
